# Effect of Physical Activity during Pregnancy on the Risk of Hypertension Disorders and Gestational Diabetes: Evidence Generated by New RCTs and Systematic Reviews

**DOI:** 10.3390/jcm13082198

**Published:** 2024-04-11

**Authors:** Cristina Taliento, Irene Piccolotti, Arianna Sabattini, Mara Tormen, Rosaria Cappadona, Pantaleo Greco, Gennaro Scutiero

**Affiliations:** 1 Department of Medical Sciences, Obstetrics and Gynecology Unit, University Hospital “Sant’Anna”, 44121 Ferrara, Italy; cristina.taliento@unife.it (C.T.); irene.piccolotti@unife.it (I.P.); arianna.sabattini@unife.it (A.S.); mara.tormen@unife.it (M.T.); rosaria.cappadona@unife.it (R.C.); g.scutiero@ospfe.it (G.S.); 2 Department of Development and Regeneration—Woman and Child, KU Leuven, 3000 Leuven, Belgium; 3 Department of Medical Sciences, Institute of Obstetrics and Gynecology, University of Ferrara, Via Fossato di Mortara, 64/B, 44121 Ferrara, Italy

**Keywords:** physical activity, pregnancy, hypertension disorders, pre-eclampsia, gestational diabetes

## Abstract

Hypertensive disorders of pregnancy (HDP) and gestational diabetes mellitus (GDM) are the most common medical complications in pregnancy. Physical exercise is considered to be safe and beneficial during pregnancy. Moreover, pregnancy could be considered as an opportunity for healthcare providers to promote positive lifestyle behavior and optimize the well-being of pregnant women. Since there are few up-to-date reviews evaluating the role of exercise and the risks of developing obstetrical complications, we performed a review to investigate the effects of physical activity and exercise during pregnancy compared to a control group, focusing on the risk of development of HDP and GDM. We searched Medline and Web of Science, including only randomized controlled trials (RCTs) and systematic reviews. This review supports a beneficial effect of exercise and provides evidence that it significantly decreases the risk of HDP and GDM.

## 1. Introduction

Several studies have reported the benefits of physical activity and exercise during pregnancy, which include reduced risk of excessive gestational weight gain, decreased risk of gestational diabetes, and preeclampsia [1,2,3,4,5,6,7,8]. Evidence also indicates that exercise during pregnancy lowers rates of preterm births and decreases the risk of caesarean section and postpartum length of stay, length of labor, and delivery complications. In addition, physical activity during pregnancy reduces depressive disorders and improves wellbeing. Moreover, it is known to influence the cardiovascular physiology of pregnancy by increasing heart rate, cardiac output, and ventilation [4,5].

After initial hesitation on the part of obstetric care providers, the general acceptance of exercise in pregnancy has grown in the past decade, and recent studies continued to support the notion that exercise during pregnancy is safe for the mother and the fetus [6]. ACOG recommends that in the absence of obstetric or medical contraindications, pregnant women should be encouraged to engage in regular, moderate-intensity physical activity during pregnancy. This also involves educating women about the risks and benefits of exercise during pregnancy and writing an individualized exercise program for each woman [9].

Hypertensive disorders of pregnancy (HDP) and gestational diabetes mellitus (GDM) are two of the leading causes of maternal morbidity and mortality. Although exercise during pregnancy is considered to be beneficial and safe for pregnant women, there is limited high-quality evidence in the literature examining the effect of physical activity on mitigating the risk of gestational hypertension and GDM. Moreover, discrepancies between the results of the available studies may be accounted for by differences in study design, study population, and methods for ascertaining exercise characteristics of the study population.

In this narrative literature review, we examine and discuss evidence of the benefits of prenatal activity, generated by new systematic review and counseling, on physical activity during pregnancy, according to the main national guidelines (ACOG 2020, WHO, American College of Sports Medicine), and then we focus on the risk of HDP and GDM in relation to the level of physical activity during pregnancy.

## 2. Materials and Methods

The search included articles published in the MEDLINE and Web of Science databases. Keywords used were: ‘physical activity’ OR ‘physical exercise’, ‘pregnancy’ OR ‘gestation’, ‘hypertension disorders’, ‘gestational hypertension’, ‘cardiovascular adaptations’, ‘pre-eclampsia’, ‘gestational diabetes’, ‘physical activity recommendations’ AND ‘pregnancy’.

We included 11 studies published over a period of 18 years (from 2004 to 2022). The characteristics of the included studies are presented in Table 1.

No additional statistical processing of the material was conducted.

The aim of this article was to review the current state of knowledge on the effects of physical activity on pregnancy outcomes to promote overall health and fitness during pregnancy.

## 3. Results

### 3.1. Physical Activity and Risk of Hypertension Disorders in Pregnancy

HDP represent the major medical challenges, affecting between 10% and 15% of pregnancies worldwide. Every year, around 70,000 mothers and 500,000 babies die because of HDP [10]. The incidence of HDP continues to increase as a result of advanced age at first pregnancy, increased prevalence of obesity, and other cardiometabolic risk factors.

Hypertension is defined as a systolic blood pressure equal or greater than 140 mmHg or diastolic blood pressure equal to or greater than 90 mmHg [11]. The expression of the disease is mediated through endothelial dysfunction and increased systemic resistance, resulting in hypertension with a highly variable degree of organ dysfunction [12].

HDP can lead to growth restriction, oligohydramnios, placental abruption, preterm birth, and perinatal death. Elevated systolic blood pressures in pregnancy, even when below the threshold for diagnosing gestational hypertension, are also linked to preterm delivery, newborn small for gestational age (SGA), and low birth weight [13]. Furthermore, women with a history of hypertension in pregnancy are also at increased risk for cardiovascular disease later in life [14]. The life expectancy of women who developed preeclampsia during pregnancy is reduced, on average, by 10 years, due to the risk of cardiovascular and cerebrovascular events. So far, no effective treatment is available other than antihypertensive drugs and termination of pregnancy for the most severe forms [11].

Lifestyle changes before and during pregnancy is considered to be safe and beneficial to pregnant women and may reduce both maternal and fetal risks. Physical activity could play a role in lowering blood pressure and improving cardiovascular fitness in expectant mothers. It may also reduce the risk of preeclampsia, reducing the maternal concentration of oxidative substances and protecting the endothelium from damage from oxidative stress [15]. The American College of Obstetricians and Gynecologists recommends moderate-intensity physical exercise every day or at least three times a week during pregnancy [9]. Malosso et al., in their study, compared pregnant women assigned before 23 weeks to an aerobic exercise regimen with pregnant sedentary women. Women who were randomized in early pregnancy to aerobic exercise (30–60 min 2–7 times a week) had a significantly lower incidence of HDP (5.9% vs. 8.5%; relative risk (RR) 0.70, 95% confidence interval (CI) 0.53–0.83; seven studies, 2517 participants), specifically, a lower incidence of gestational hypertension (2.5% vs. 4.6%; RR 0.54, 95% CI 0.40–0.74), while the incidence of preeclampsia was similar (2.3% vs. 2.8%; RR 0.79, 95% CI 0.45–1.38; six studies, 2230 participants). Moreover, the incidence of cesarean delivery was decreased by 16% in the exercise group (19.6% vs. 21.5%; RR 0.84, 95% CI 0.73–0.98) [16] (Table 2).

In addition, Kawasara et al. studied the effect of physical exercise on the development of preeclampsia. This systematic review included 17 studies: 6 case–control studies, 10 prospective cohort studies, and 1 randomized clinical trial. The case–control studies and the randomized study showed a protective role of exercise on the development of preeclampsia (OR 0.77; CI 0.64–0.91 and OR 6.34; 95% CI 0.72–55.37, respectively), the prospective cohort studies showed no significant difference. Specifically, the analysis of the 6 case–control studies showed that exercise had a protective effect of around 23% on the development of preeclampsia [17] (Table 2). However, Vollebregt et al. conducted a population-based prospective cohort study, which found that playing sports or total leisure time physical activity at a high level did not show any significant association with preeclampsia (OR 0.43; 95% CI 0.17–1.10) or gestational hypertension (OR 0.78; CI 0.36–1.69) in nulliparous women [18]. Literature regarding this subject is sometimes conflicting. Studies often use different methods and definitions, which makes it difficult to compare the results. In this study, Vollebregt et al. evaluated the role of exercise in leisure time, while more vigorous activity seems to be associated with a lower incidence of preeclampsia (Table 2).

Al-Huda et al. assessed the cardiorespiratory fitness, measured as VO_2_ max or peak, VO_2_ at anaerobic threshold, or work rate at peak VO_2_. Assuming that exercise has been shown to improve maternal cardiorespiratory fitness (CRF), this study showed an important relationship between CRF and risk of hypertension and preeclampsia, suggesting a protective role of exercise. According to the study, CRF can identify women at risk for HDP. Before pregnancy, women who developed hypertension had lower CRF than those without hypertension (VO_2_ max < 37 vs. >37 mL O_2_/min). VO_2_ max at 14–18 weeks of pregnancy was lower among women who developed preeclampsia vs. normotensive women (three studies, 275 women; mean difference 0.43 mL/kg/min [95% CI 0.97–0.10]). In the postpartum group, a similar trend was observed with lower VO_2_ peak in women with previous preeclampsia (three studies, 208 women; 0.26 mL/kg/min [−0.54, 0.02]) [19] (Table 2). Moreover, Danielli at al. provide information about the most effective ways to exercise. They included data about women performing supervised exercise during pregnancy and compared them to a control group. The likelihood of developing hypertension was notably decreased in the intervention group compared to the control groups, with an estimated pooled cumulative incidence of developing hypertension of 3% in the intervention groups and 5% in the control group (95% CI: 0.40–0.72, *p* < 0.001). Specifically, engaging in both aerobic and anaerobic exercises or solely practicing yoga was associated with a greater beneficial effect compared with performing aerobic exercise only (mixed-OR: 0.50, 95% CI: 0.33–0.75, *p* < 0.001; yoga-OR: 0.28, 95% CI: 0.13–0.58, *p* < 0.001; aerobic exercise only-OR: 0.87, 95% CI: 0.55–1.37, *p* < 0.539). This review showed a beneficial role of either structured exercise or yoga [20] (Table 2). Regarding prenatal diet, physical activity, and behavior, Oteg–Ntin et al. found that lifestyle interventions are associated with a trend toward decreased incidence of GDM among overweight and obese populations. However, no clear differences were observed for other outcomes, such as large for gestational age, birth weight, or macrosomia [21].

Furthermore, in a meta-analysis published in 2018, Devenport et al. analyzed evidence from 34 RCTs (9755 participants) regarding the association between prenatal exercise and HDP. The meta-analytic effect indicated 19% lower odds of HDP with exercise compared with no exercise (OR 0.81, 95% CI 0.65–1.00). Exercise-only interventions reduced the odds of developing HDP by 41% (OR 0.59, 95% CI 0.37–0.94) [7] (Table 2).

#### Physical Activity and Risk of Gestational Diabetes and/or Macrosomia

Approximately 2% to 5% of pregnant women are estimated to have pre-existing or GDM, with up to 0.4% of women in the UK and 0.9% of pregnant women in the USA having pre-existing diabetes (type 1 or type 2) [22,23].

GDM manifests as high blood glucose (hyperglycemia) occurring for the first time or being first recognized during pregnancy. The prevalence of GDM ranges from 1% to 14% among pregnant women, with certain groups being at a high risk, such as women who are overweight or obese, older, of specific ethnicities, have had GDM previously, or have a family history of type II diabetes [24]. GDM can lead to significant complications for both mothers and infants including macrosomia, hypoglycemia, erythema, hypocalcemia, jaundice, and birth trauma. Moreover, children are predisposed to obesity, abnormal glucose tolerance, and the onset of diabetes during adolescence or early adulthood, contrasting with offspring of normoglycemic women [25]. Women diagnosed with GDM had higher probabilities of induced labor, cesarean delivery, and preterm birth. Furthermore, there are potential long-term complications for both mothers and infants, including an increased risk of developing type II diabetes [26].

Certain dietary patterns, such as those characterized by low fiber and high glycemic load, along with physical inactivity, represent modifiable risk factors for GDM [27]. Research indicates that lifestyle interventions targeting diet and exercise modifications within the general population have the potential to prevent type II diabetes. It is posited that similar interventions may also contribute to the prevention of GDM during pregnancy.

To date, studies of the influence of physical activity on reducing GDM risk have been inconclusive. In a previous Cochrane review performed by Han et al., results from three RCTs with a moderate risk of bias suggested no significant difference in GDM incidence between women who were physically active during pregnancy [28]. A second Cochrane review by Bain et al., including 13 RCTs, showed that no clear difference was observed in the risk of developing GDM for women receiving a combined diet and exercise intervention compared with women receiving no intervention [27].

In a large prospective RCT including 300 singleton women, patients allocated to the exercise group were assigned to exercise 3 times per week (at least 30 min/session) until 37 weeks of gestation. Wang et al. found that women in the exercise group had significantly lower odds of GDM (22.0% vs. 40.6%; *p* < 0.001) [29] (Table 3). Consistent with this, Dempsey et al. considered the risk of GDM in relation to typical daily activities performed during the year before pregnancy and during pregnancy [30]. Regular participation in any recreational physical activity during the year before and/or during the first 20 weeks of the index pregnancy was associated with an approximate halving of the risk of GDM in this case-control study; women reporting any activity during this period experienced a 55% reduction in the risk of GDM (OR = 0.45; 95% CI 0.28–0.74). This risk reduction was further increased for women who received an additional exercise intervention during both of these time periods. Indeed, women who engaged in physical activity before and during pregnancy experienced a 69% reduced risk (RR = 0.31; 95% CI: 0.12–0.79). Furthermore, the number of hours spent performing recreational activities and the energy expended were related to a decrease in risk of GDM; women spending ≥ 4.2 h/week engaged in physical activity experienced a 76% reduction in GDM risk (RR = 0.24; 95% CI: 0.10–0.64). The authors also found that women who climbed stairs, irrespective of their participation in recreational physical activities and pre-pregnancy body mass index, also experienced a reduction in the risk of GDM [30] (Table 3).

Similarly, Doi et al. evaluated the efficacy of physical activity for GDM prevention in high-risk women. The results of this review demonstrated that physical activity programs reduced the risk of GDM (RR 0.69; 95% CI 0.51–0.94). The pooled effect across in-facility exercise was (RR 0.51; 95% CI 0.37–0.71) [31] (Table 3). In another systematic review and metanalysis, Russo et al. determined a 28% lower risk of GDM among patients assigned to a physical activity intervention compared with those in a control group (RR 0.72; 95% CI 0.58–0.91). These results are consistent with findings from observational research [32,33] (Table 3).

In another meta-analysis of 8 cohort studies comprising 72,694 participants, Pastorino et al. concluded that physical activity in late, but not early, pregnancy is consistently associated with a lower risk of large-for-gestational-age (LGA) fetuses and macrosomia, but not SGA [34] (Table 3).

The association between prenatal exercise and the risk of developing GDM was also examined in the meta-analysis performed by Devenport et al., which included 46 RCTs (n = 14,923). Although the authors defined the quality of evidence as ‘low’ because of the serious risk of biases, results showed that prenatal exercise was associated with 24% lower odds of developing GDM compared with no exercise (OR 0.76; 95% CI 0.65–0.88). Moreover, as discussed previously, a significant difference was observed between sedentary women and active women before pregnancy in terms of the risk of pre-eclampsia [7] (Table 3).

## 4. Discussion

The benefits of physical activity during pregnancy are receiving more and more interest from the world of research, and the scientific literature on the subject is constantly increasing. Evidence has emerged suggesting that physical activity from the first trimester decreases the odds of GDM, pre-eclampsia, gestational hypertension, and excessive gestational weight gain [9].

HDP and GDM are the most frequent obstetric disorders during pregnancy. These clinical conditions are characterized by similar risk factors, such as obesity, insulin resistance, advanced maternal age, and excessive gestational weight gain [35]. Moreover, in [36], they were found to share similar pathophysiological mechanisms that included inflammation, vascular dysfunction, oxidative stress, and vascular disease.

In this regard, recent studies underline the role of physical activity on angiogenesis [37]. Morland et al., studying the benefits of exercise on brain health, identified the lactate receptor HCAR1 as a key regulator of VEGF and angiogenesis in the brain in response to physical activity. In support of the role of exercise in the prevention of diabetes and hypertension through endothelial protection, Piani et al., in their systematic review, highlight the role of new susceptibility genes such as cluster of differentiation (CD) 93 in the pathogenesis of vascular damage, which has been shown to underlie hypertensive and metabolic disorders [38]. In a clinical setting, the Hyperglycemia and Adverse Pregnancy Outcome (HAPO) study found positive associations between increasing plasma glucose levels and the incidence of preeclampsia [25]. Similarly, in a retrospective study including 1813 patients, results showed that pregestational BMI (OR 2.3; 95% CI 1.16–2.30) and severity of GDM (OR 1.7; 95% CI 1.21–2.38) were independently and significantly associated with an increased risk of preeclampsia [39].

Thus, although many studies have documented a strong association between maternal BMI, pre-eclampsia, and GDM, the physiopathological aspect underlying these conditions is not yet fully understood [40,41,42]. Proinflammatory cytokines have been pointed out as among the factors that could support that link. Many studies showed that adipose tissue releases adipokines that, by inducing proinflammatory cytokines (leptin, IL-1β, IL-6, and TNF-α), generated an inflammatory environment that is considered a partial causative factor in the pathogenesis of several metabolic, inflammatory, and neoplastic diseases [43]. The anti-inflammatory cytokines (adiponectin, IL-4, IL-10, IL-13, and perhaps IL-6) attenuate inflammation by restricting cytokine production, up-regulating their antagonist-binding proteins, and suppressing inflammatory activity. Satoh et al. first demonstrated that abnormalities in the leptin-to-adiponectin ratio were observed in patients diagnosed with impaired glucose tolerance and hypertension [44].

A nested case–control study that included 198 GDM cases and 192 controls showed that women with GDM had a higher baseline leptin concentration compared to those without GDM [45]. Consistent with these results, another study demonstrated that plasma leptin was higher (*p* < 0.001) in women with GDM than in women with normal glucose tolerance [46].

A similar correlation was observed between high levels of proinflammatory cytokines and pre-eclampsia. Taylor et al., in a large study including 746 pregnant women, found that leptin concentrations were significantly higher in women with preeclampsia (*p* = 0.0117) and term preeclampsia (*p* = 0.0228) compared with controls [47]. Moreover, many studies showed how higher levels of leptin were found in the placentas of women with GDM and hypertension disorders.

In the literature, there is growing evidence that exercise increases secretion of anti-inflammatory adipokines and reduces proinflammatory cytokines [48,49,50]. The GESTAFIT Project aimed to analyze the influence of an exercise-training program on inflammatory markers in maternal venous and arterial and venous cord serum. The exercise group followed a 60 min 3 days/week aerobic-resistance exercise training from the 17th gestational week to delivery. Results showed that in the exercise group, there was a statistically significant decrease in TNF-α concentrations (*p* = 0.02) compared to the control group. Moreover, the exercise group was associated with a significant increase in anti-inflammatory cytokines such as IL-1β (*p* = 0.03) and IL-10 (*p* = 0.05) compared to the control group [51].

In the absence of specific complications, ACOG, Canadian guidelines, and RCOG encouraged, during pregnancy, types of physical activity that are now notoriously safe during pregnancy, such as stationary cycling, aerobic exercises, dancing, resistance exercises, stretching, hydrotherapy, and water aerobics [9,52,53].

Several new systematic reviews (SRs) evaluated the association between physical activity and HDP, GDM, or birth size. The presence of SRs on the subject, characterized by the robustness of the methodology, allows for a more accurate level of evidence, and increases the impact of the studies on clinical practice. Moreover, meta-analysis methodologies allow the pooling of results across studies. However, not all the SRs have the same characteristics; some included only RCTs (Magro–Malosso, 2017; Danielli, 2022; Bain, 2015; Suhail, 2020; Cai, 2020), while others also included observational studies, providing a lower level of evidence (Kasawara, 2012; Al-Huda, 2022; Russo, 2015; Pastorino, 2018).

Furthermore, only one systematic review (Suhail, 2020) evaluated the certainty of the evidence with the GRADE method, as recommended by the Cochrane Handbook for Systematic Reviews of Interventions [Higgins]. One of the principal limitations cited by the systematic review is that the primary studies varied in duration, frequency, type, and length of exercise programs, as well as among the methods used to assess physical activity, including the diagnostic thresholds for GDM. The same heterogeneity is retrieved in the prevalence of confounding factors, such as smoking, employment, parity, BMI, and history of prior GDM. Additionally, some of the primary studies were based on self-reported physical activity, with the possibility of recall biases, and not all questionnaires used were validated. In conclusion, our review confirms the need for further studies of high methodological quality on the subject, with more structured and standardized physical activity programs, to better compare the results between the studies. In addition, there is a need to obtain more data stratified for the various comorbidities of the patients, for the intensity of physical activity, and for the different types of physical exercise.

## 5. Conclusions

Physical activity is associated with a reduced risk of GDM and PE, representing a milestone for the prevention of these pregnancy complications.

Exercise during pregnancy has a protective effect against HDP by influencing the pathogenetic mechanisms and acting on associated risk factors. An adequate diet and engagement in physical activity also influence modifiable risk factors for GDM. In addition, a balanced diet and adequate physical activity are associated with a reduction in the incidence of type II diabetes mellitus.

Physicians should encourage women to adopt a more active lifestyle during pregnancy. Additionally, it is advisable for women with an elevated BMI to maintain a balanced diet in the preconception period as well as sustain a good level of physical activity during pregnancy.

## Figures and Tables

**Table 1:** Articles included in the search.

Author Year	Study Design	Sample Size	Time Period
Magro- Malosso, 2017	Meta-analysis	5075 patients	From the inception of each database to February 2017
Kasawara, 2012	Systematic review	231 articles, 214 of which were excluded, 17 remained.	No limitation to year of publication (up to June 2011)
Vollebregt, 2010	Prospective cohort study	3679 nulliparous women	Between January 2003 and March 2004
Al-Huda, 2022	Systematic review and meta analysis	14 studies (2406 women)	No date restriction was applied
Danielli, 2022	Systematic review and meta analysis	16 randomized controlled trials	From the inception of each database to November 2021
Devenport, 2018	Systematic review and meta analysis	46 randomized controlled trials	Between 2002 and 2017
Wang, 2017	Randomized clinical trial	300 women	Between December 2014 and July 2016
Dempsey, 2004	Prospective cohort study	909 women	Between April 1998 and February 2001
Doi, 2020	Meta-analysis	1467 adult women in 11 eligible trials	After 1996 till June 2020
Russo, 2015	Systematic review and meta-analysis	10 randomized studies	Between 1966 and August 2014
Pastorino, 2019	Individual level meta-analysis	8 population-based studies comprising 72694 participants.	No date restriction was applied

**Table 2:** Physical activity and risk of hypertension disorders in pregnancy.

Author Year	Study Design	Sample Size	Intervention	Result
Magro- Malosso, 2017	Meta-analysis	5075 patients	30–60 min of aerobic exercise two to seven times per week until at least week 35 or up to delivery	Women who were randomized in early pregnancy to aerobic exercise for about 30–60 min two to seven times per week had a significantly lower incidence of gestational hypertension and cesarean delivery.
Kasawara, 2012	Systematic review	231 articles, 214 of which were excluded, 17 remained.	PA as any voluntary bodily movement that increased energy expenditure above the basal level	This systematic review indicates a trend toward a protective effect of physical activity in the prevention of pre-eclampsia.
Vollebregt, 2010	Prospective cohort study	3679 nulliparous women	Physical activity in leisure time in the past week	Playing sports or total leisure time physical activities at a high level lacked a significant association with preeclampsia and gestational hypertension
Al-Huda, 2022	Systematic review and meta analysis	14 studies (2406 women)	CRF (VO_2_ max or peak, VO_2_ at anaerobic threshold, or work rate at peak VO_2_) in women with and without HDP.	The study showed an important relationship between cardiorespiratory fitness and risk of HDP.
Danielli, 2022	Systematic review and meta analysis	16 randomized controlled trials	Supervised exercise during pregnancy	Beneficial effect of either structured exercise or yoga for preventing the onset of HDP.
Devenport, 2018	Systematic review and meta analysis	46 randomized controlled trials	Prenatal physical exercise	Lower odds of gestational hypertension in the exercise group

**Table 3:** Physical activity and risk of gestational diabetes and/or macrosomia.

Author Year	Study Design	Sample Size	Intervention	Result
Wang, 2017	Randomized clinical trial	300 women	Regular exercise in early pregnancy to prevent GDM	Cycling exercise initiated early in pregnancy and performed at least 30 min, 3 times per week, is associated with a significant reduction in the frequency of gestational diabetes mellitus in overweight/obese pregnant women
Dempsey, 2004	Prospective cohort study	909 women	Recreational physical activity before and during pregnancy	Maternal physical activity may contribute to substantial reductions in GDM risk. This advantage is greater for more intense physical activities.
Doi, 2020	Meta analysis	1467 adult women in 11 eligible trials	Physical activity in women at high risk before the 20th week of gestation	Physical activity reduced the risk of GDM compared with usual care.
Russo, 2015	Systematic review and meta analysis	10 randomized studies	Group or individual exercise interventions	Reduced risk of developing GDM in the intervention group compared with the control group.
Pastorino, 2019	Individual level meta-analysis	8 population-based studies comprising 72694 participants.	Maternal physical activity in early and late pregnancy	Late gestation maternal physical activity was inversely associated with birth weight, large for gestational age, macrosomia, and ponderal index.
Devenport, 2018	Systematic review and meta analysis	46 randomized controlled trials	Prenatal physical exercise	Lower odds of developing GDM in the exercise group.

## Data Availability

The authors confirm that the data supporting the findings of this study are available within the article.
